# Traditional circumcision during manhood initiation rituals in the Eastern Cape, South Africa: a pre-post intervention evaluation

**DOI:** 10.1186/1471-2458-8-64

**Published:** 2008-02-19

**Authors:** Karl Peltzer, Ayanda Nqeketo, George Petros, Xola Kanta

**Affiliations:** 1 Health Systems Research Unit, Social Aspect of HIV/AIDS and Health, Human Sciences Research Council, Pretoria, South Africa; 2 Department of Psychology, University of the Free State, Bloemfontein, South Africa; 3 Impilo ya Bantu Health, Lusikisiki, South Africa

## Abstract

**Background:**

Circumcisions undertaken in non-clinical settings can have significant risks of serious adverse events, including death. The aim of this study was to test an intervention for safe traditional circumcision in the context of initiation into manhood among the Xhosa, Eastern Cape, South Africa.

**Methods:**

Traditional surgeons and nurses registered with the health department were trained over five days on ten modules including safe circumcision, infection control, anatomy, post-operative care, detection and early management of complications and sexual health education. Initiates from initiation schools of the trained surgeons and nurses were examined and interviewed on 2^nd^, 4^th^, 7^th ^and 14^th ^day after circumcision.

**Results:**

From 192 initiates physically examined at the 14th day after circumcision by a trained clinical nurse high rates of complications were found: 40 (20.8%) had mild delayed wound healing, 31 (16.2%) had a mild wound infection, 22 (10.5%) mild pain and 20 (10.4%) had insufficient skin removed. Most traditional surgeons and nurses wore gloves during operation and care but did not use the recommended circumcision instrument. Only 12% of the initiates were circumcised before their sexual debut and they reported a great deal of sexual risk behaviour.

**Conclusion:**

Findings show weak support for scaling up traditional male circumcision.

## Background

Accounts of serious complications or adverse events after adolescent and adult circumcision in traditional settings in Africa are legion. Every circumcision season there are articles in national and local newspapers depicting in words and pictures cases of advanced infection, severe loss of blood, mutilation, and even deaths due to events attributable to male circumcision. In the scientific literature, there are reports listing adverse events from traditional circumcision generated from hospital records [1-5] or circumcision/initiation school inspections [6], but without knowing the total number of males circumcised in that area (i.e., the denominator), it is not possible to estimate rates of adverse events. Circumcisions undertaken in non-clinical settings can have significant risks of serious adverse events, including death. Among 50 patients admitted to hospital with post-circumcision complications in Nigeria and Kenya between 1981 and 1998, 80% had been circumcised by medically untrained traditional surgeons. One of these patients died from septicaemia, two lost their penis from gangrene, and five others had permanent disability from complete or partial amputation of the glans or shaft [5]. A further study of 48 boys presenting to hospital with post-circumcision complications in Nigeria found that the commonest complication was haemorrhage (52% patients) and infection (21%) [3]. Among the Babukusu ethnic group in western Kenya, circumcision is part of the initiation rite for youth aged 8–20, and circumcision may be carried out traditionally or medically by a doctor, clinician or health professional. More detailed examination of 298 of the boys at 45–96 days post-operation showed that traditional circumcision was also associated with slower healing, more swelling, laceration and keloid scarring [7]. Among the Xhosa in South Africa, an unsterilized unwashed blade may be used on a dozen or more initiates in a single session [2,8]. Initiates are also significantly dehydrated during their 2 week period of seclusion in the belief that this reduces weeping of the wound, and after-care may be in the hands of a traditional attendant with no basic medical training [2]. The combination of dehydration and septicaemia can result in acute renal failure, gangrene, tetanus or even death [2,8]. The Eastern Cape provincial Department of Health recorded 2262 hospital admissions, 115 deaths and 208 genital amputations for circumcisions between 2001 and 2006 [9]. To address this, traditional surgeons are now required by law to be officially recognized and registered with the provincial Department of Health [2]. The Eastern Cape Legislature promulgated a law, known as Application of Health Standards in Traditional Circumcision Act No. 6 of 2001, which regulates traditional male circumcision [10].

Little information is available concerning rates of complications in traditional settings in Africa [7] and this seems the first study to test the feasibility of a safe traditional male circumcision intervention.

## Methods

### Design

Pre-post training intervention assessment of traditional male circumcision of initiates attending 17 initiation schools in two Local Service Areas (LSAs), Nyandeni and Qaukeni of the O.R.Tambo district, Eastern Cape province.

### Sample and procedure

The sample included 160 Xhosa initiates, mean age 18.7 years (SD = 1.9), range16 to 26 years; 27 (16.9%) were below 18 years; the number of years of formal education completed was mean 8.1 years (SD = 2.6), range 2 to 14 years.

Initiates were first informed about the study when undergoing medical examination for circumcision. Two junior HSRC researchers contacted traditional surgeons who had previously been trained and had also formally consented to the study about their circumcision schedule. At the second day after circumcision the designated medical officer, the clinical research nurse and an HSRC researcher visited the initiation school to introduce the study and individual formal consent was taken from the initiates about physical examination and an interview by the research nurse and the HSRC researchers. The clinical research nurse physically examined all initiates in each initiation school at the 2^nd^, 4^th^, 7^th ^and 14^th ^day after circumcision. In addition, they were interviewed with a semi-structured questionnaire on the 7^th ^day after circumcision by the HSRC researcher. Complications identified at examination were either treated by the clinical nurse or referred to a health facility. Permission had been obtained from traditional surgeons and nurses to conduct examinations and an interview with their initiates. The clinical male nurses had been trained in circumcision physical examination by XK. Recruitment began on June 2007, and follow-ups were completed by mid July, 2007; all initiates agreed to participate in the study. The research protocol was reviewed and approved by the Human Sciences Research Council (HSRC) research ethics committee protocol REC 1/17/05/06. The provincial health department of the Eastern Cape, the district health office and traditional authorities in the study areas approved the study.

### Intervention

The traditional surgeons and nurses from which the initiates were recruited had undergone a five day training by XK and GP including modules on: Introduction into initiation rites; Traditional Community Regulation as well as statutory regulation of Traditional Male Circumcision and Initiation into Manhood; Structure and function of the male sex organs; Procedure of safe circumcision, Infection control; STIs/STDs; HIV/AIDS; Infection control measures; Aftercare of the initiate including after care of the circumcision wound and initiate as a whole; Detection and early management of common complications of circumcision; Nutrition and fluid management; Code of conduct and ethics for traditional health practitioners; and Sexual health education [6]. Traditional surgeons were also provided with a tool box including surgical (scalpel) blades, scalpel handles, latex hand gloves, sterilization instruments, and paper towel rolls, and traditional nurses received also the tool box including latex hand gloves, sterilization instruments, and paper towel rolls. Details on the training, the manual and its evaluation are reported elsewhere [11].

### Measure

The assessment of initiates included a physical examination of the operation area following a standardized index of adverse reactions of male circumcision including pain, bleeding, haematoma, swelling, wound infection, delayed wound healing, excessive skin removed, insufficient skin removed, problems with urination and problems with appearance [12,13].

The interview schedule for the initiates included socio-demographics, sexual and HIV risk behaviour and expectations about circumcision, based on a literature review [6,14-16]. Response options included mostly "yes" and "no" responses.

In addition, four items of the Body Parts Satisfaction Scale by Berscheid, Walster, and Bohrnstedt [17] were used. Individual items indicated respondents' level of satisfaction with aspects of their body ranging from 1 = extremely dissatisfied to 6 = extremely satisfied.

### Data analysis

The SPSS (version 14.0) statistical programme was used to analyze the data with descriptive statistics.

## Results

### Adverse events following surgery

From 192 initiates physically examined at the 14th day after circumcision by a trained clinical nurse 40 (20.8%) had mild delayed wound healing, 31 (16.2%) had a mild wound infection, 22 (10.5%) mild pain and 20 (10.4%) had insufficient skin removed. It was also be observed that (1) swelling or haematoma decreased from 9.9% on the 2^nd ^day to 2.1% on the 14^th ^day and (2) mild bleeding decreased from 4.6% on the 2^nd ^day to 1.0% on the 14^th ^day (see Table 1).

**Table 1:** Circumcision complications of initiates assessed at four time points in percent

**Medical Complication**	**Description of Medical Complication**	**Severity**	**2^nd ^day N = 152 (%)**	**4^th ^day N = 159 (%)**	**7^th ^day N = 193 (%)**	**14^th ^day N = 192 N(%)**
**Pain**	3 or 4 on pain scale	Mild	13.2	26.4	21.2	22 (11.5)
	5 or 6 on pain scale	Moderate		5.0		
	7 on pain scale	Severe				
**Excessive bleeding**	Dressing/bandage soaked through with blood at a routine follow-up visit	Mild	4.6	1.9	3.1	2 (1.0)
	Bleeding that requires a special visit by the attendant for medical attention.	Moderate				
	Bleeding that requires a visit to hospital or clinic and surgical re-exploration to control	Severe				
**Infection**	Erythema (redness of skin) more than one cm beyond incision	Mild	16.4	13.8	20.3	31 (16.2)
	Purulent discharge (pus) from wound	Moderate			1.6	
	Cellulitis (infection spreading under the skin) or wound necrosis (gangrene or black, dead tissue) requiring hospitalization	Severe				
**Excessive skin removed**	Client concerned, but there is no discernable abnormality	Mild	2.0	1.9	1.6	1 (0.5)
	Skin is tight, but additional operative work not necessary	Moderate				
	Requires re-operation or transfer to a medical facility to correct the problem	Severe				
**Insufficient kin removed**	Foreskin partially covers the head only when extended	Mild	13.2	14.4	17.1	20 (10.4)
	Foreskin still partially covers the head and re-operation is required to correct	Moderate				
	Not applicable	Severe				
**Swelling or haema-toma (collection of blood)**	More swelling than usual, but no significant discomfort	Mild	9.9	4.4	2.6	4 (2.1)
	Significant tenderness and discomfort; no surgical procedure needed or only minor surgical re-exploration required	Moderate				
	Surgical re-exploration or visit to hospital or clinic required to correct	Severe				
**Damage to the penis**	Mild bruising or injury, not requiring treatment	Mild				
	Bruise or injury to the head or shaft of the penis requiring pressure dressing or additional surgery to control or repair	Moderate	0.6	0.6	0.5	1 (0.5)
	Portion or all of the head or shaft of the penis severed	Severe				
**Delayed wound healing**	Healing takes longer than usual, but no extra treatment necessary	Mild	2.0	11.9	16.6	40 (20.8)
	Additional non-operative treatment required	Moderate				
	Requires re-operation to correct or visit to clinic or hospital required	Severe				
**Problems with passing urine**	Temporary complaints that resolves without treatment	Mild	1.3	1.9	2.1	2 (1.0)
	Requires special return to the clinic, but no additional treatment required	Moderate				
	Requires referral to another facility for management	Severe				
**Dehydration**	Severe thirst, but passed urine in past 24 hours and no dizziness	Mild	5.3	5.7	7.8	7 (3.6)
	Severe thirst, and becoming light-headed, with no urine in past 24 hours	Moderate				
	No urine in past 24 hours and lost consciousness or required visit to hospital or clinic to provide fluids or to treat related medical problems, such as kidney failure	Severe				
**Appearance**	Client concerned, but no discernable abnormality	Mild	2.6	3.8	0.5	1 (0.5)
	Significant wound disruption or scarring, but does not require re-operation	Moderate				
	Requires re-operation	Severe				

### Infection control

Initiates were asked on the 7^th ^day after circumcision about circumcision procedures. Most (85%) indicated that the traditional surgeon had been wearing gloves when performing circumcision and two-thirds (69%) of the traditional nurses wore gloves when caring. Further, 53% of the initiates reported that they had been circumcised with an *assegai *(spear) and 47% indicated that they had been circumcised with a surgical blade or knife.

## Expectations about traditional male circumcision

When participants were asked about their perceptions about traditional circumcision most respondents 126 (70%) felt that they expected some complication following male circumcision.

Most (57.8%) expected to stay in the bush for a month, 40% less than a month and 11.1% for more than a month.

Participants were asked questions relating to their body satisfaction and the outcomes are that the level of satisfaction to all participants were high; 72.9% reported that they were extremely satisfied, 18.8% reported that they were quite satisfied with 5.6% reported their dissatisfaction with the appearance of their sex organs (see Table 2).

**Table 2:** Body parts satisfaction post-circumcision (7th day)

	Chest		Size of sex organs		Appearance of sex organs		Overall body appearance	
	N	%	N	%	N	%	N	%

Extremely satisfied	146	80.2	142	78.5	132	72.9	156	86.2
Quite satisfied	28	15.4	28	15.5	34	18.8	19	10.5
Somewhat satisfied	3	1.6	2	1.1	5	2.8	3	1.7
Somewhat dissatisfied	1	0.5	3	1.7	2	1.1	0	0
Quite dissatisfied	0	0	1	0.6	1	0.6	1	0.6
Extremely dissatisfied	4	2.2	5	2.8	7	3.9	2	1.1

### Sexual behaviour and HIV risk

Most initiates (88%) ever had sexual intercourse, the mean age of first sex was 14.8 years (SD = 2.4), range 10 to 25 years, 69% had had sex when they were less than 18 years old, 55% had been sexually active in the past 12 months, 29% reported that they had sexual intercourse with two partners and twenty-four (15%) had sexual intercourse with three and more sex partners in their life time. Only 38% indicated that they had used a condom with their last sexual partner, 9% had received an STD diagnosis in the past 12 months, 15% used alcohol in the past week and 10% indicated to have sex under the influence of alcohol. Almost all had received AIDS training, most felt knowledgable about HIV and most did not feel susceptible to HIV (see Table 3).

**Table 3:** Sexual behaviour and HIV risk of initiates

	N	%
Sex		
Ever sex	142	87.6
First sex with 17 years and below	111	68.5
Had sex in past 12 months	97	55.1
Number of sex partners		
1	54	33.8
2	47	29.0
3 and more	24	14.8
Condom use at last sex	64	38.1
During the past 12 months has a doctor or other health professional told you that you had a sexually transmitted disease (STD)	15	8.5
Past month alcohol use	34	19.4
Past week alcohol use	27	15.4
Sex under the influence of alcohol	19	10.9
How susceptible/at risk to get HIV		
Very susceptible	21	11.5
Susceptible	11	6.0
Neutral	40	22.0
Not susceptible	43	23.6
Not susceptible at all	67	36.8
Self-rated HIV knowledge		
Very poor	21	11.6
Poor	39	21.5
Average	45	24.9
Good	19	10.5
Very good	57	31.5
Did you previously receive AIDS education	159	97.8

## Discussion

Using a pre-post intervention evaluation design, this study evaluated 192 initiates who had undergone a traditional male circumcision intervention with trained traditional surgeons and traditional nurses in the Eastern Cape, South Africa. The study found that from 192 initiates physically examined at the 14th day after circumcision by a trained clinical nurse the following major adverse events following surgery were found: 40 (20.8%) had mild delayed wound healing, 31 (16.2%) had a mild wound infection, 22 (10.5%) mild pain and 20 (10.4%) had insufficient skin removed. Bailey and Egesah [7] conducted an examination of 298 boys at 45–96 (M = 62) days post-operation following traditional circumcision in Kenya and found in 21.4% not fully healed, foreskin remaining 11.6%, swelling 13.9%, lacerations 16.9% and keloid scarring 17.4%. When insufficient foreskin is excised, the results are uncosmetic and cicatrisation of the distal foreskin and wound contracture may occur. Sepis due to infection occurs in up to 10% of patients and varies in severity from mild and local to heavy suppuration and ulceration or to septicaemia in which case it may lead to death if not treated early with antibiotics [2]. The 1989 review of the American Academy of Pediatrics' Task Force on Circumcision reported that the rate of postoperative complications of male circumcision was approximately 0.2% to 0.6% in the United States. The majority of complications were minor, the most common being local infection and bleeding [18]. Krieger et al. [19] found among 479 medically circumcised male adults in Kenya that 3.5% were associated with adverse events judged definitely, probably or possibly related to the procedure. The most common adverse events were wound infections (1.3%), bleeding (0.8%), and delayed wound healing or suture line disruption (0.8%). After 30 days, 99% reported being very satisfied with the procedure.

This study found that only 12% were to be circumcised before their sexual debut and reported a great deal of sexual risk behavior: 9% had been diagnosed with a sexually transmitted infection in the past 12 months, 15% reported to have had three or more sex partners in their lives and 38% had not used a condom at last sex. Similar HIV risk behaviour was also found in a study by Jewkes et al. [20] among sexually active Xhosa youth. Shisana and Simbayi [21] also found in the 2002 national HIV prevalence survey in South Africa that most men only get circumcised after they have become sexually active. In this study among 21% initiates delayed wound healing was found 14 days post-operation. Most young men (88%) were found to be sexually active prior to circumcision and such a long period for healing could expose them to elevated risk for HIV infection through an open wound. Bailey and Egesah [7] found that in their post-operative traditional male circumcision sample in Kenya that the wounds of 24% of those circumcised had not fully healed when they were observed at 60 days post-operation.

Relatively high rates of circumcision complications were found among this sample of initiates who underwent traditional circumcision with specifically trained traditional surgeons and nurses. Although traditional surgeons had been trained and were provided with surgical blades prior to conducting the studied circumcisions, more than half (53%) had used the culturally more acceptable but less safer *assegai *(spear) for the circumcision, which may have contributed to some of the circumcision complications. Regarding wound care 31% of the traditional nurses did not wear gloves that had been provided during the training which may have contributed to wound infections.

## Conclusion

It appears that a five day training for traditional surgeons and nurses is not sufficient and that more training is needed in the surgical procedure, the control of sepsis, post-operative wound care, recognition of complications, and referral to hospital would also be beneficial. Further supportive trainings may be the most effective way for promoting cognitive, attitudinal, and behavioural change. The use of the appropriate surgical instruments and wound care needs to be emphasized by traditional leaders. In order to improve timely and appropriate monitoring of initiates by designated medical officers initiation schools should only be established in more central and easily accessible locations. Post operation counselling with initiates should be conducted including HIV risk reduction, reproductive, HIV pre-test and manhood counselling.

Traditional surgeons and nurses need to be appropriately registered and fulfilling all criteria stipulated in the male circumcision act. Consideration should be given to a certification process for practitioners who undergo approved training programmes [7]. A training manual for traditional male circumcision (explicating traditional methods of circumcision and wound care) should be further tested and finalized. Additional studies are needed of complication rates and male circumcision practices with specifically trained traditional surgeons and nurses in order to design the appropriate intervention for the safest traditional male circumcision possible.

Still one should emphasize the danger of the procedure, even with an intervention of additional training. Improving the quality of male circumcision services could reduce healing times and thus reduce risk of HIV infection in those who resume sexual activity soon after circumcision. Counselling males not to engage in sex until they are fully healed must be included in post-op instructions. Circumcision cannot be a stand alone procedure; it must be integrated with behavioural and reproductive health counselling in order to minimize both complications and risk of HIV infections [7].

## Competing interests

The author(s) declare that they have no competing interests.

## Authors' contributions

KP conceptualized and designed the study, analysed and interpreted the data, drafted and revised the manuscript. AN and GP participated in the design of the study, data collection, and data analysis. XK participated in the design of the study and the training of the traditional surgeons and nurses. All authors read and approved the final draft of the manuscript.

## Pre-publication history

The pre-publication history for this paper can be accessed here:


